# Supplementary material for: The Urinary Exosomal miRNA Expression Profile is Predictive of Clinical Response in Lupus Nephritis
Source: Int J Mol Sci. 2020 Feb 18;21(4):1372. doi: 10.3390/ijms21041372 (PMC7073236; doi:10.3390/ijms21041372)
Supplement: Supplementary file 1 [file ijms-21-01372-s001.pdf]

Supplementary Material

1. Supplementary Methods

1.1. Patients and Samples

Renal disease was defined by either a total urinary protein level ≥ 0.5 g/day, an increment of serum creatinine levels of more than 0.5 mg/dL, or the presence of active sediment by microscopic examination, and confirmed by renal biopsy. Nephrotic syndrome was defined as a proteinuria > 3.5 g/24 h and a serum albumin < 30 g/L. Disease activity was assessed by the Systemic Lupus Erythematosus Disease Activity Index 2000 update (SLEDAI-2Ks; range 0–105) [1]. Renal activity was defined as the sum of the SLEDAI-2Ks accrued in the renal domain of the measure (rSLEDAIs; range 0–16) and defined by a SLEDAI-2K = 4) [2]. Accumulated damage was assessed by the Systemic Lupus International Collaborating Clinics Damage Index (SLICC-DI) (rSDI; range 0–47) [3]. As a part of the protocol of SLE renal involvement study, renal biopsy is performed when renal involvement is suspected. Renal biopsies were examined by light and immunofluorescence microscopy and categorized according to the International Society of Nephrology/Renal Pathology Society Classification (ISN/RPS) [4] and rated for activity (AI) and chronicity (CI) [5].

All patients received intravenous (i.v) methyl-prednisolone (500 mg × 3 days) followed by a tapering dose of oral prednisone along with at least a 24-month course of oral mycophenolate mofetil (MMF), as previously reported, with cyclophosphamide or tacrolimus as induction and maintenance therapy. Patients were classified as responders or non-responders. Responders (R) were defined as those patients who achieved complete remission (CR), defined as urinary protein excretion < 0.3 g/24 h, normal urinary sediment, and normal or stable renal function (within 10% of normal estimated glomerular filtrate rate if previously abnormal) [6]. Non-responders (NR) included those in partial remission (defined as proteinuria > 0.3 but < 3.5 g/24 h or a decrease in proteinuria by at least 50% from the initial value and < 3.5 g/24 h), and those with an increase in serum creatinine concentration ≥ 0.6 mg/dL and a creatinine clearance > 15% below the baseline value.

On the day of the serum sample collection, key laboratory measurements were also obtained, including white blood cells (WBCs), serum creatinine, estimated glomerular filtrate rate (eGFR), serum albumin, complement levels (C3 and C4), anti-double-stranded DNA (anti-dsDNA), and 24-h protein excretion. Patients with urinary tract infection, diabetes mellitus, pregnancy, malignancy, and non-lupus-related renal failure were excluded.

1.2. Urinary Exosome Isolation

Exosomes were extracted from fresh urine samples using the miRCURY™ Exosomes Isolation Kit (Exiqon, Woburn, MA, USA). Briefly 10-mL samples were centrifuged at 3200× *g* for 5 min to remove debris and stored at 4 °C. After adding 4 mL of precipitation buffer B to the supernatant, the samples were newly incubated at 4 °C for 60 min, and again centrifuged for 30 min at 3200× *g*, removing the supernatant. Exosomes were extracted from remaining pellet. The pellet was resuspended in 100 µL of resuspension buffer to obtain intact exosomes and stored at −80 °C prior to RNA isolation.

1.3. Serum Exosome Isolation

Isolation exosomes were obtained using the exoRNeasy Serum/Plasma Kit (Qiagen, Hilden, Germany) from 1-mL pre-centrifuged serum samples (16,000× *g* for 10 min at 4 °C). One volume of Buffer XBP was added to the serum sample. Mix serum:Buffer XBP was then added to the exoEasy spin column and spun for 1 min at 500× *g*. The flow-through was discarded and 3.5 mL of Buffer XWP were added. This was then spun for five min at 5000× *g*, discarding the flow-through together

with the collection tube. The spin column with the containing extracellular vesicles was then transferred into a new collection tube and kept at −80 °C until characterization.

1.4. Exosome Characterization by Western-Blot, Cryo-TEM, and NanoSight Analysis

The protein isolation of exosomes was carried out using the Total Exosome RNA & Protein Isolation Kit (Applied Biosystems, Foster City, CA, USA). For Western blot analysis, the samples were loaded on 10% polyacrylamide gels and transferred to the membrane. Different antibodies against exosomal markers (rabbit anti-CD9, rabbit anti-AQP2, rabbit anti-TSG101, dilutions 1:1000, Abcam, Cambridge, UK) were used to incubate the membrane and the proteins were detected using enhanced chemiluminescence detection reagents according to the manufacturer's instructions (Thermo Fischer, Waltham, MA, USA).

The characterization by cryo-TEM and NanoSight was done at the Universitat Autonoma de Barcelona (UAB, Barcelona, Spain). For cryo-TEM characterization, a drop of a dilution of exosome pellet (1:100 in PBS) was put on a frozen grid. This was transferred to a cryopreparation chamber (Leica EM CPC, Barcelona, Spain) using a GATAN cryotransfer apparatus to prepare the sample for freezing with propane and ethane. The temperature of the grid was maintained at −174 °C. The vitrified samples were examined using a JEM-1400 electron microscope (Jeol, Peabody, MA, USA) with an acceleration voltage of 40–120 kV. For NanoSight characterization, different dilutions of exosomes were examined using the Nanosight LM-20 particle size analyzer (Malvern Panalytical, Malvern, UK).

1.5. RNA Extraction from Urinary Exosomes and Serum Exosomes

For urinary exosomes, the lysis process was performed by adding 350 μL of lysis solution and 200 μL of 95% ethanol to exosome samples. Lysate with ethanol was applied onto the column and centrifuged at 14,000× g for one minute. Purification was improved by adding 400 μL of wash solution to the column, with centrifuging at 14,000× *g* for 1 min three times. Finally, the purified RNA bound to the column was obtained by adding 50 μL of elution buffer with brief centrifugation at 200× *g* for 2 min for uniform distribution into the column. Later, centrifugation for 1 min at 14,000× *g* was necessary to obtain the purified RNA samples. These were stored at −80 °C. The degree of RNA quality of different samples was evaluated using Bioanalyzer PicoChip (Agilent Technologies, Santa Clara, CA, USA).

For serum exosomes, QIAzol (700 μL) was added to serum exosome dilution and immediately spun for 5 min at 5000× *g*; the lysate was collected and transferred completely into a new 2-mL tube. The lysate was incubated for 5 min at room temperature (15–25 °C). Then, 90 μL of chloroform were added and incubated after vigorous shaking for 3 min at room temperature. Then, the mixture was centrifuged at 12,000× *g* at 4 °C for 15 min. The upper aqueous phase was then transferred to a new collection tube, avoiding transferring any interphase. Two volumes of 100% ethanol were added and mixed. Then, 700 μL of the new sample were pipetted into an RNeasy MinElute spin column and centrifuged at 10,000× *g* for 15 s at room temperature. The step was repeated until completing volume transference. Once completed, the sample was spun for 15 s at 10,000× *g* after adding 700 μL of RWT buffer. One 15-s centrifugation cycle of 500 μL buffer RPE was then applied and another of 2 min was performed afterward. Then, the RNeasy MinElute spin column was centrifuged at full speed with an open lid to completely dry the membrane. Finally 17 μL of RNase-free water were directly added to the center of the spin column membrane; after letting it stand for 1 min, the sample was centrifuged for 1 min at full speed to elute the purified RNA. Samples were then stored at −80 °C for later use.

1.6. MicroRNA Screening with Pre-Designed Plates

A reaction mixture of 6 μL of diluted cDNA (1:80), 594 μL of free-nuclease water, and 600 μL of PCR Master Mix (Exiqon, Woburn, MA, USA) was applied to each well in a 10-μL volume. The plate was later sealed with optical sailing and briefly spun (1500× *g* for 1 min) to collect the samples. Real-time PCR amplification was performed following an initial cycle of polymerase activation for

10 min at 95 °C. The amplification process consisted of 45 amplification cycles of 10 s at 95 °C and 1 min at 60 °C. Finally a dissociation cycle was applied, consisting of 15 s at 95 °C , followed for 20 s at 60 °C and finally 15 s at 95 °C. Transcription was measured by using ABI PRISM 7000 (Thermo Fisher, Waltham, MA, USA).

1.7. miRNA Screening Analysis

Data were obtained from the Exiqon MicroRNA arrays were analyzed by the Vall d'Hebron Research Institute bioinformatic unit. The microarray statistical analysis was performed using the R statistical software package (www.R-project.org) [7] and the libraries developed for microarray data analysis by the Bioconductor Project (www.bioconductor.org) [8]. The biological relationship of the results obtained in the microarray was studied using the Ingenuity Pathway Analysis (IPA®, QIAGEN Redwood City, CA, USA, www.qiagen.com/ingenuity).

1.8. Validation of miRNA Expression by qPCR-RT

Initially a first-strand cDNA synthesis reaction was performed to provide a template for all microRNA real-time PCR assays using the miRCURY LNA™ Universal RT microRNA PCR (Exiqon, Woburn, MA, USA). Each RNA sample was normalized to a 5 ng/µL concentration using nuclease-free water. Then, 2 µL were used in combination with a mix of 2 µL of reaction buffer + 1 µL of enzyme mix + 5 µL nuclease-free water. The reaction was vortexed gently, ensuring the thorough mixing of all reagents. The resulting solution was later incubated for 60 min at 40 °C followed by a heat-inactivation of reverse transcriptase for 5 min at 95 °C. Immediately the samples of cDNA were stored at −80 °C. To avoid process variability the final samples used for RT-qPCR arrays were obtained by repeating the previous steps three-fold and mixing the extracts. All cDNA samples were diluted 1:6.6 in nuclease-free water. Every well contained a combination of 5 µL of PCR master mix, 1 µL of PCR primer mix, and 4 µL of diluted cDNA. All analysis were repeated in triplicate. The required amount of primer:master mix solution was calculated and prepared in advance when multiple real-time PCR reactions were performed with same microRNA primer. Fifteen percent of all reagents were additionally included to compensate for pipetting excess material. Real-time PCR amplification was performed following an initial cycle of polymerase activation for 10 min at 95 °C. The amplification process consisted of 45 amplification cycles of 10 s at 95 °C and 1 min at 60 °C. Finally a dissociation cycle was applied consisting of 15 s at 95 °C, followed by 20 s at 60 °C and finally 15 s at 95 °C. Transcription was measured by using a ABI PRISM 7000. Data were normalized based on the expression of the least variable miRNA from the microarray study (miR-423) and relative expressions were calculated using the $2^{-\Delta\Delta Ct}$ Livak method [9].

1.9. Double Fluorescent in situ hybridization (ISH) Detection

The *miRCURY LNA™ microRNA ISH Optimization Kit* (Exiqon®) was used for visualizing gene expression and microRNA localization in renal tissue samples. Samples were obtained from histopathological laboratory bank at the Hospital Vall Hebron, and used through a deparaffinized method by placing slides in xylene and successively lower ethanol concentration solutions. MicroRNAs were demasked by using Proteinase-K. The Proteinase-K method allows the access of the labelled LNA™ probes into the cell and hybridization with the corresponding RNA sequence. Then, 100 µL of proteinase-K/proteinase-K Buffer Mix were applied to fully cover every slide of tissue, followed by incubation for 10 min at 37 °C. Later, a wash was performed by placing the slides into PBS, followed by a dehydrating process in new ethanol solutions with increased ethanol concentrations.

The hybridization mix was made by mixing a dilution of microRNA ISH buffer 1:1 with RNase-free water with the LNA™ microRNA probe at a 1:650 concentration. A concentration of 1:500 LNA™ U6 snRNA was used as a positive control. The probe concentration was optimized for an optimal microRNA ISH signal using the provider's instructions. The *Hybridization Mix* was then

applied and kept at 50 °C for 120 min. Tissue sections were later washed in saline-sodium citrate (SSC) buffer at decreasing concentrations. First, a 5-min SSC wash at hybridization temperature was performed, followed by a 10-min SSC (5x) wash at hybridization temperature, a 10-min SSC (0.2x) wash at hybridization temperature, and finally a 10-min SSC (0.2x) wash at room temperature (RT). Before detection, the hybridization tissue section was incubated for 15 min at RT using 3% H_2O_2 to block endogenous peroxidases before applying horseradish peroxidase-conjugated antibodies to avoid high background signaling due to intrinsic tissue peroxidase activity. Also, autofluorescence quenching was applied by using 20-min incubation on 0.5% Sudan Black with 70% ethanol. Thereafter, the slides were washed three times (3 min per wash) with TN buffer (0.1 M Tris-HCL, pH 7.5, 0.15 M NaCl). The next step consisted of a 30-min incubation with *blocking buffer* (10 mL TN buffer + 0.5 g BSA + 200 µL sheep serum) at RT. After that, *blocking solution* was removed and a primary antibody solution (anti-DIG/HRP or anti-FITC/HRP) was applied with a dilution 1:400 in blocking buffer. After a 30-min incubation period, 5-min TNT buffer (0.1 M Tris-HCL, pH 7.5, 0.15 M NaCl, 0.3% (v/v) Triton X-100) washes were applied three times. Tyramide signal amplification (TSA) technology (Exiqon, Woburn, MA, USA) was used to improve and amplify the fluorescent signal. Then, 10 min of incubation with 500 µL of TSA Working Solution, a dilution 1:50 of TSA Plus Stock Solution, and amplification diluent were applied. Later, the slides were washed three times, at 5 min per wash, with TNT buffer in the dark with shaking. The last steps were then repeated, applying the other primary antibody (anti-FITC/HRP instead of anti-DIG/HRP or vice versa). Prior visualization a 10-s DAPI (4′,6-diamidino-2-phenylindole) application was done to visualize the cellular nucleus. Finally, an Olympus BX61 motorized upright microscope with fluorescence and phase optics for immunofluorescence imaging was used to visualize all tissue sections.

1.10. Cell Cultures

Primary human renal mesangial cells (MsCM), renal proximal tubular epithelial cells (HRPTEpiC), and Primary Human renal endothelial cells (HRGEC) were purchased from InnoProt (Derio, Spain) and cultured in the recommended media provided by the manufacturer. Cells were grown at 37 °C in a humidified 5% CO_2 atmosphere. Cell passes were performed using TrypLE™ Express (Gibco® Thermofisher Scientific).

1.11. Immunofluorescence

For cell immunofluorescence, they were incubated with paraformaldehyde (4%) for 20 min and permeabilizated with 0.1% Triton for 10 min. Blocking using PBS 5% BSA was performed for 1 h at room temperature, and primary antibodies were incubated overnight at 4 °C. Secondary antibodies were incubated for 2.5 h at room temperature.

For renal tissue immunofluorescence, this was performed on paraffin-embedded (FFPE) renal biopsies during renal flare (n = 3 for each subgroup). Slides were baked, soaked in xylene, passed through graded alcohols, and then pre-treated with 10 mM citrate (pH 6) in a steam pressure cooker (Decloaking Chamber; BioCare Medical, Walnut Creek, CA, USA) as per the manufacturer's instructions. All further steps were done at room temperature in a hydrated chamber. Slides were then treated with peroxidase block (DAKO) for 5 min to quench endogenous peroxidase activity. Staining was performed with 1:100 rabbit anti-Hypoxia inducible factor 1 alpha (Gennetex, GTX127309, Irvine, CA, USA), rabbit anti-KLF4 (Proteintech, 11880-1-AP, Manchester, UK) or mouse anti-FOXO1 (Proteintech, 66457-1-Ig) overnight at 4 °C. Slides were then labelled with diluted 1:250 Alexa 488 donkey anti-rabbit (Abcam, ab150061, Cambridge, UK) and/or Alexa 647 goat anti-mouse (Abcam, ab150119) for 2 h at room temperature and cover slipped using Fluoromount-G with DAPI (Invitrogen, Thermo Fisher Scientific, Carlsbad, CA, USA). Finally, using an Olympus BX61 motorized upright microscope with fluorescence and phase optics for immunofluorescence imaging, all the tissue sections were visualized.

1.12. Evaluation of Hybridization in Situ and Immunofluorescence

After antibody incubation, the results were evaluated on blinded specimens by the Vall d'Hebrón Pathology Unit under the supervision of the nephropathologist (Dr. Marta Vidal, Barcelona, Spain). The percentages of cells expressing the different probes were scored semi-quantitatively as follows: 0 (no expression), 1 (11%–20%), 2 (40%–60%), or 3 (>80%). Staining intensity was scored semi-quantitatively as 0 (no staining), 1 (weakly positive), 2 (moderately positive), or 3 (strongly positive).

2. Supplementary References

1. Hochberg, M.C. Updating the American college of rheumatology revised criteria for the classification of systemic lupus erythematosus. *Arthritis Rheum.* **1997**, *40*, 1725.
2. Gladman, D.D.; Ibañez, D.; Urowitz, M.B. Systemic lupus erythematosus disease activity index 2000. *J. Rheumatol.* **2002**, *29*, 288–291.
3. Gladman, D.; Ginzler, E.; Goldsmith, C.; Fortin, P.; Liang, M.; Urowitz, M.; Bacon, P.; Bombardieri, S.; Hanly, J.; Hay, E.; et al. The development and initial validation of the systemic lupus international collaborating clinics/American College of Rheumatology damage index for systemic lupus erythematosus. *Arthritis Rheum.* **1996**, *39*, 363–369.
4. Yung, S.; Chan, T. Redefining lupus nephritis: Clinical implications of pathophysiologic subtypes. *Nat. Rev. Nephrol.* **2017**, *13*, 483–495.
5. Austin, H.A. 3rd; Muenz, L.R.; Joyce, K.M.; Antonovych, T.A.; Kullick, M.E.; Klippel, J.H.; Decker, J.L.; Balow, J.E. Prognostic factors in lupus nephritis. Contribution of renal histologic data. *Am. J. Med.* **1983**, *75*, 382–391.
6. Liang, M.; Schur, P.; Fortin, P.; St.Clair, E.; Balow, J.; Costenbader, K.; Crofford, L.; De Pablo, P.; Dooley, M.A.; Finckh, A.; et al. The American College of Rheumatology response criteria for proliferative and membranous renal disease in systemic lupus erythematosus clinical trials. *Arthritis Rheum.* **2006**, *54*, 421–432.
7. R Core Team. R: A language and environment for statistical computing. R Foundation for Statistical Computing: Vienna, Austria, 2008.
8. Gentleman, R.C.; Carey V.J.; Bates, D.M.; Bolstad, B.; Dettling, M.; Dudoit, S.; Ellis, B.; Gautier, L.; Ge, Y. Gentry, J.; et al. Bioconductor: Open software development for computational biology and bioinformatics. *Genome Biol.* **2004**, *5*, R80.
9. Livak, K.; Schmittgen, T. Analysis of Relative Gene Expression Data Using Real-Time Quantitative PCR and the $2^{-\Delta\Delta Ct}$ Method. *METHODS*. **2001**, *25*, 402–408.

3. Supplementary Figure

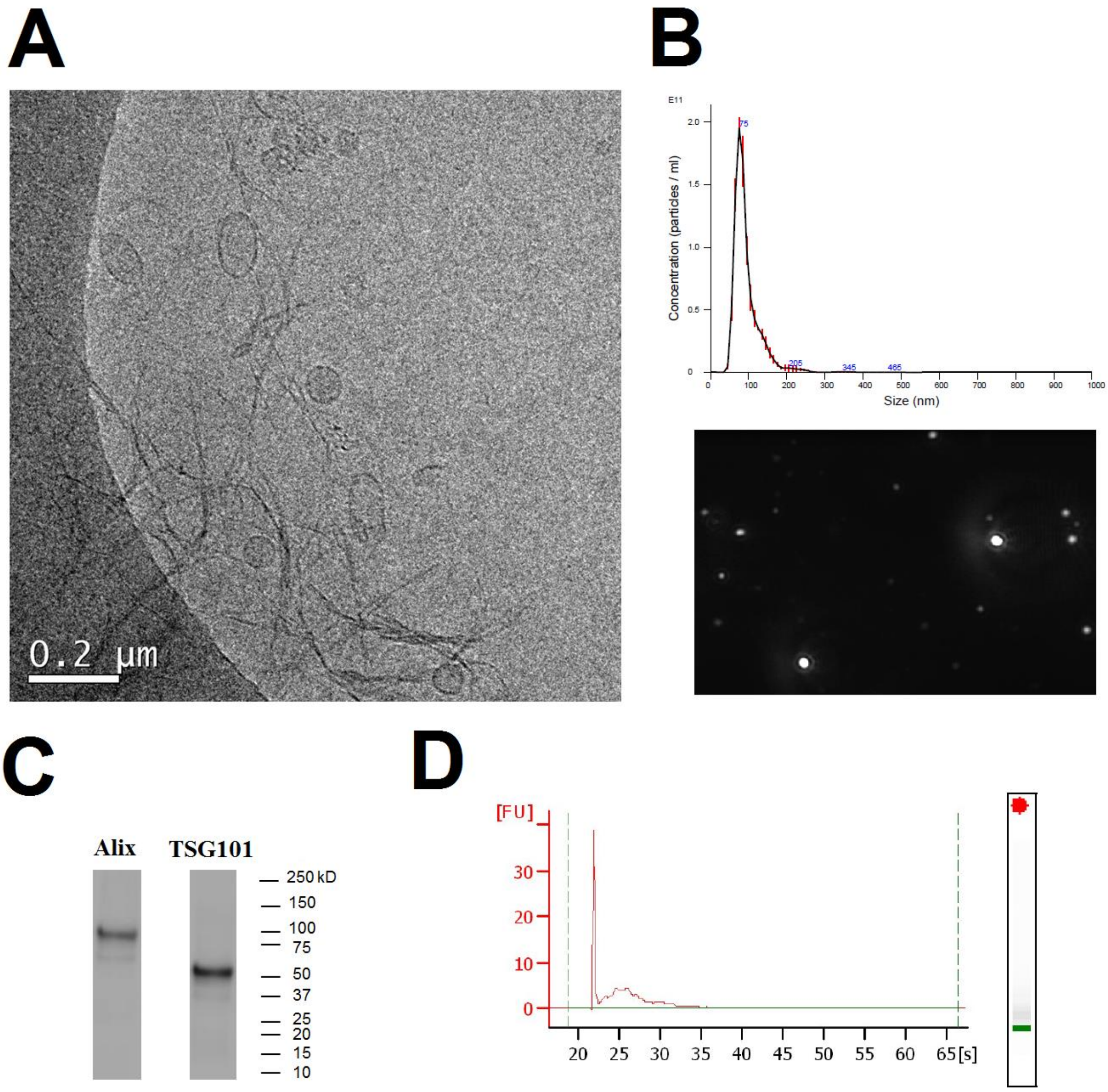

Figure S1. Characterization and identification of urinary exosomes. (**A**) Cryo-transmission electron micrograph of urinary exosomes showed small vesicles with sizes ranging from 57 to 130 nm in diameter. (**B**) Size distribution of urinary exosomes analyzed by Nanoparticle Tracking Analysis. (**C**) Western blotting of Alix protein and TSG101 characteristics of urinary exosomes. (**D**) The PicoBio analysis of RNA extracted from urinary exosomes revealed a predominant population of small RNAs (<200 nt).

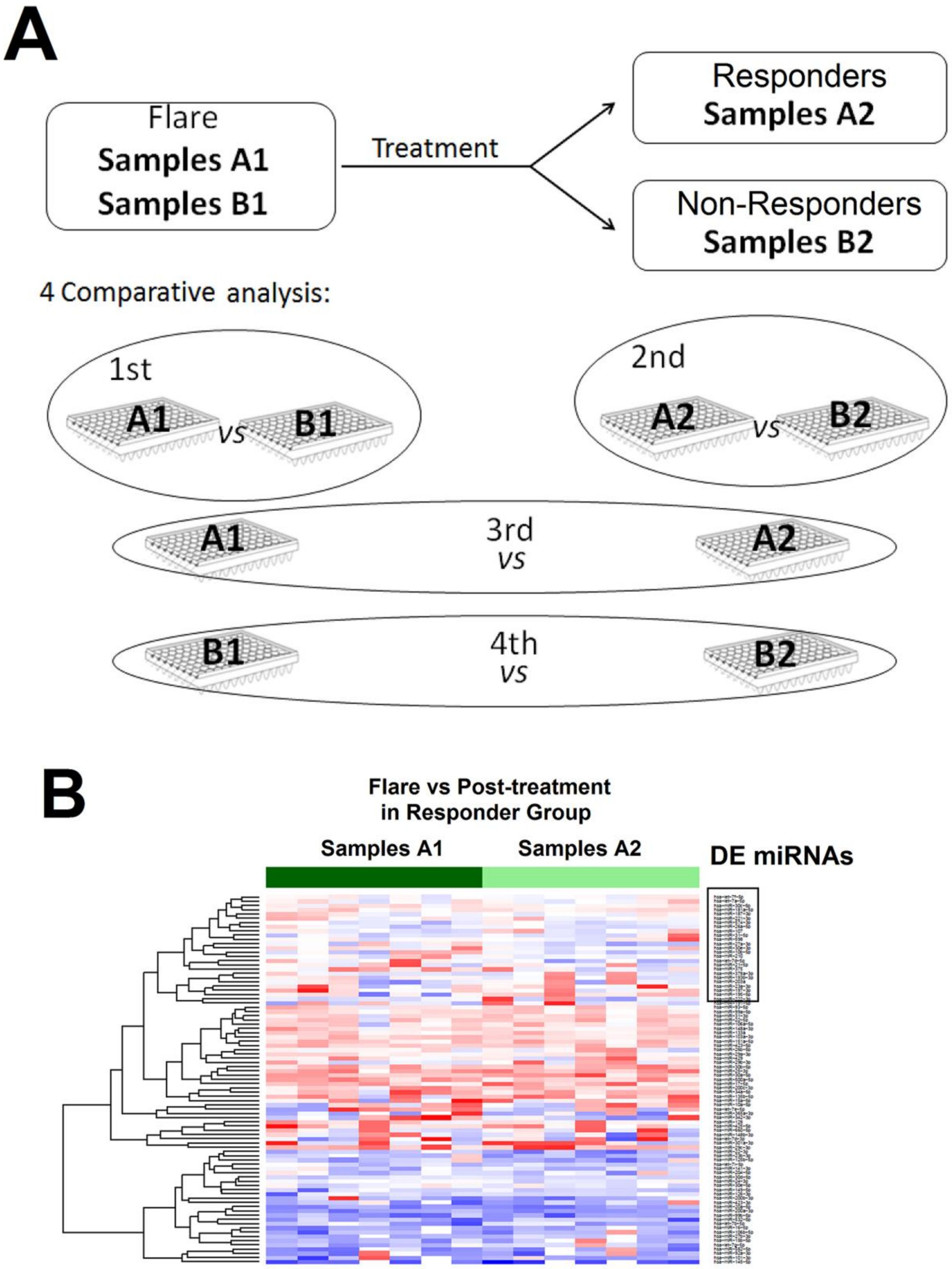

Figure S2. Comparative analysis for urinary exosomal miRNA expression according to clinical lupus nephritis (LN) response. (**A**) Scheme of the four comparative analyses performed by the Bioinformatic Unit. (**B**) Heatmap from flare and post-treatment comparison in the lupus nephritis responder group illustrating levels of significantly changed microRNA expression (fold change > 1.5 and p-value < 0.05). DE miRNAs: differentially expressed miRNAs. Color red means upregulated and color blue means downregulated in heatmap.

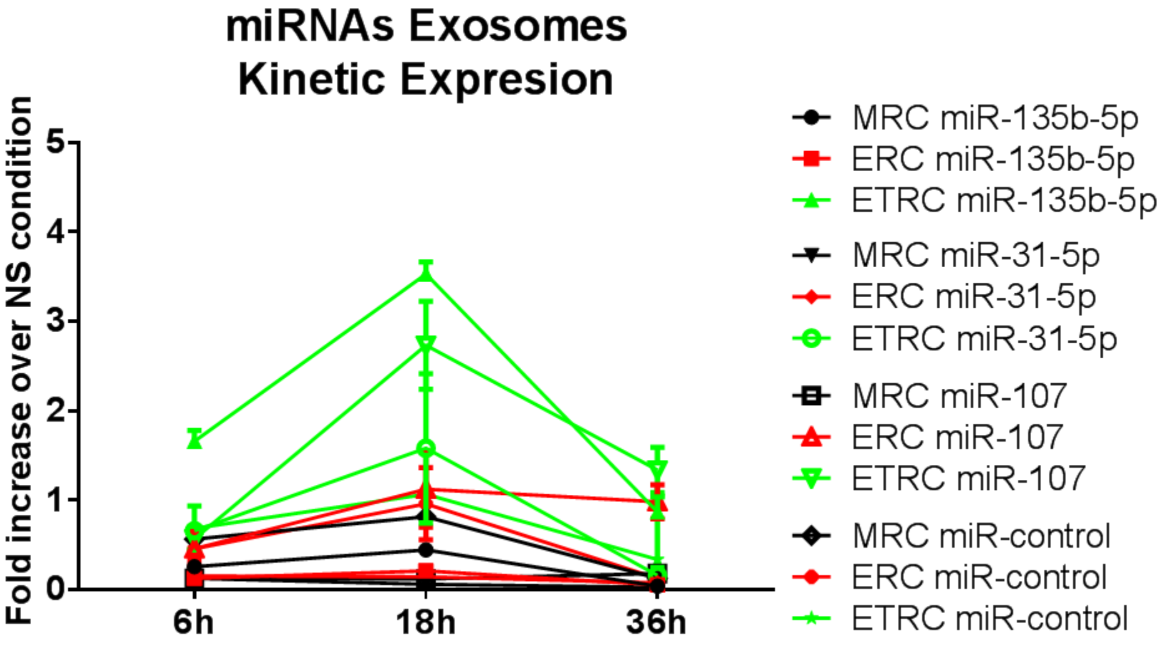

Figure S3. Kinetic study production of microRNA (miRNA) urinary exosomes. Values of miRNA expression in exosomes obtained from mesangial renal cells (MRCs), endothelial renal cells (ERCs), and epithelial tubular renal cells (ETRCs) after 6, 18, and 36 h of cytokine stimulation.

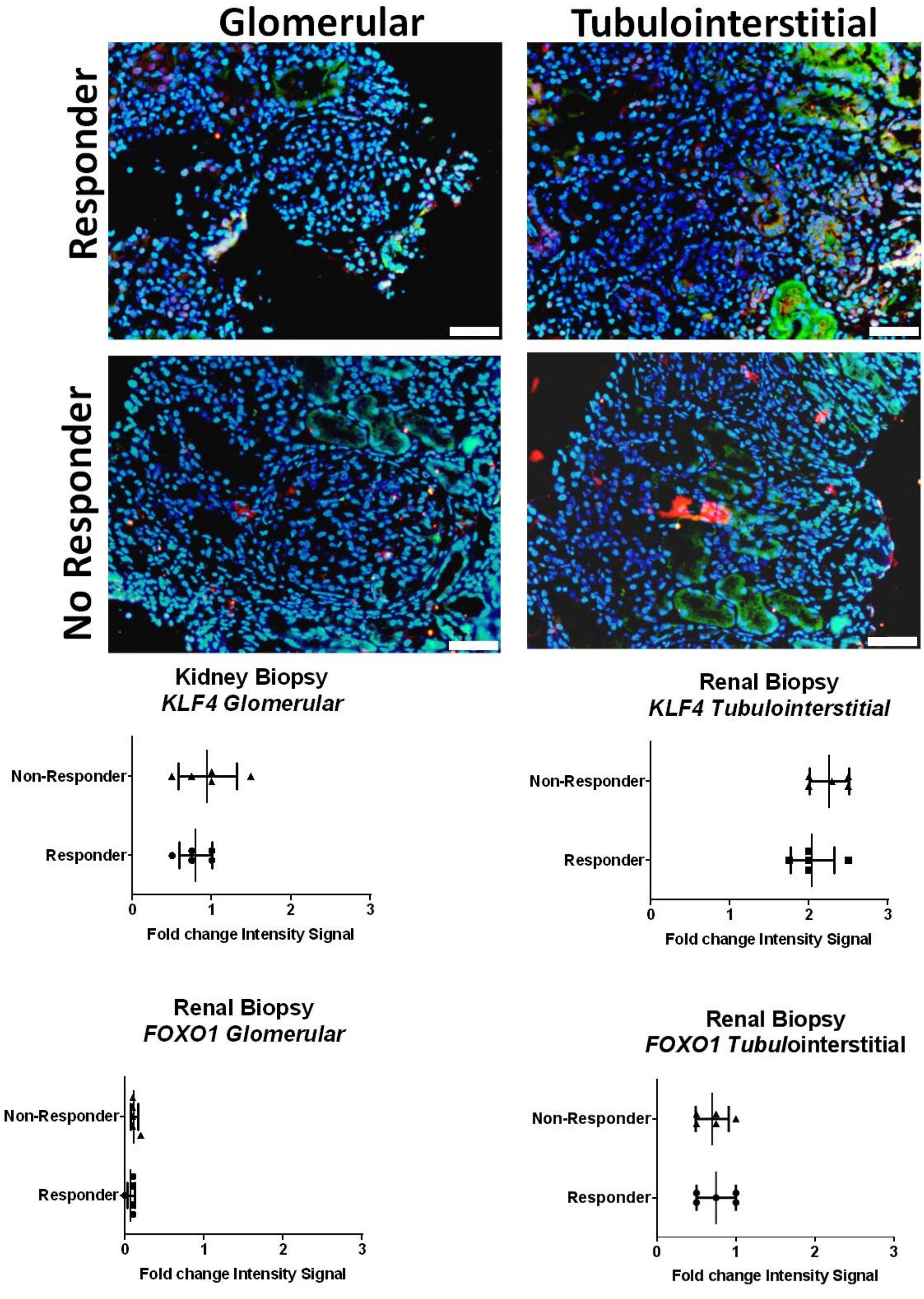

Figure S4. Immunofluorescence of kidney biopsies. Kruppel-like factor 4 (KLF4) (green) and Forkhead box protein O1 (FOXO1) (red) did not show different protein levels between responder and non-responder kidney biopsies. Scale bar = 100 μm.

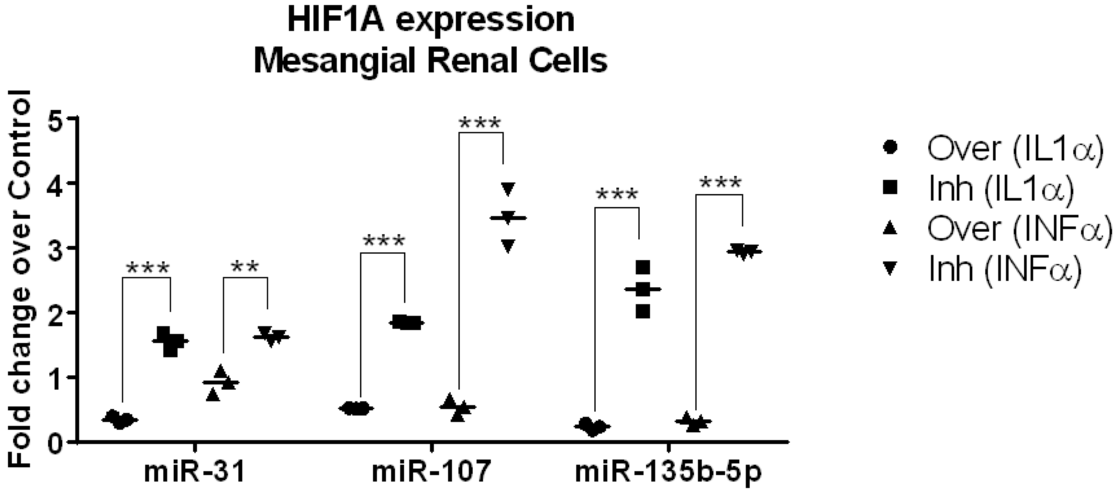

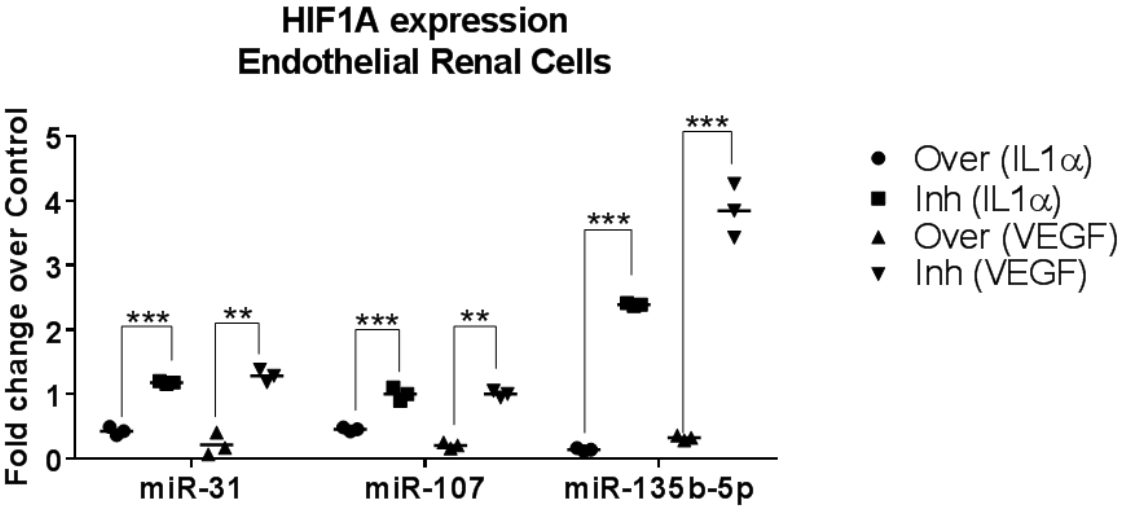

Figure S5. *HIF1A* expression levels in mesangial and endothelial renal cells. Overexpression (OVER) of miR-31, miR-107, or miR-135b-5p induced significant inhibition of HIF1A gene expression in interleukin-1 alpha, interferon alpha and vascular endothelial growth factor stimulation. In a similar way, inhibition (INH) of each miRNA induced high expression of HIF1A. *** $p < 0.0001$, ** $p < 0.001$.

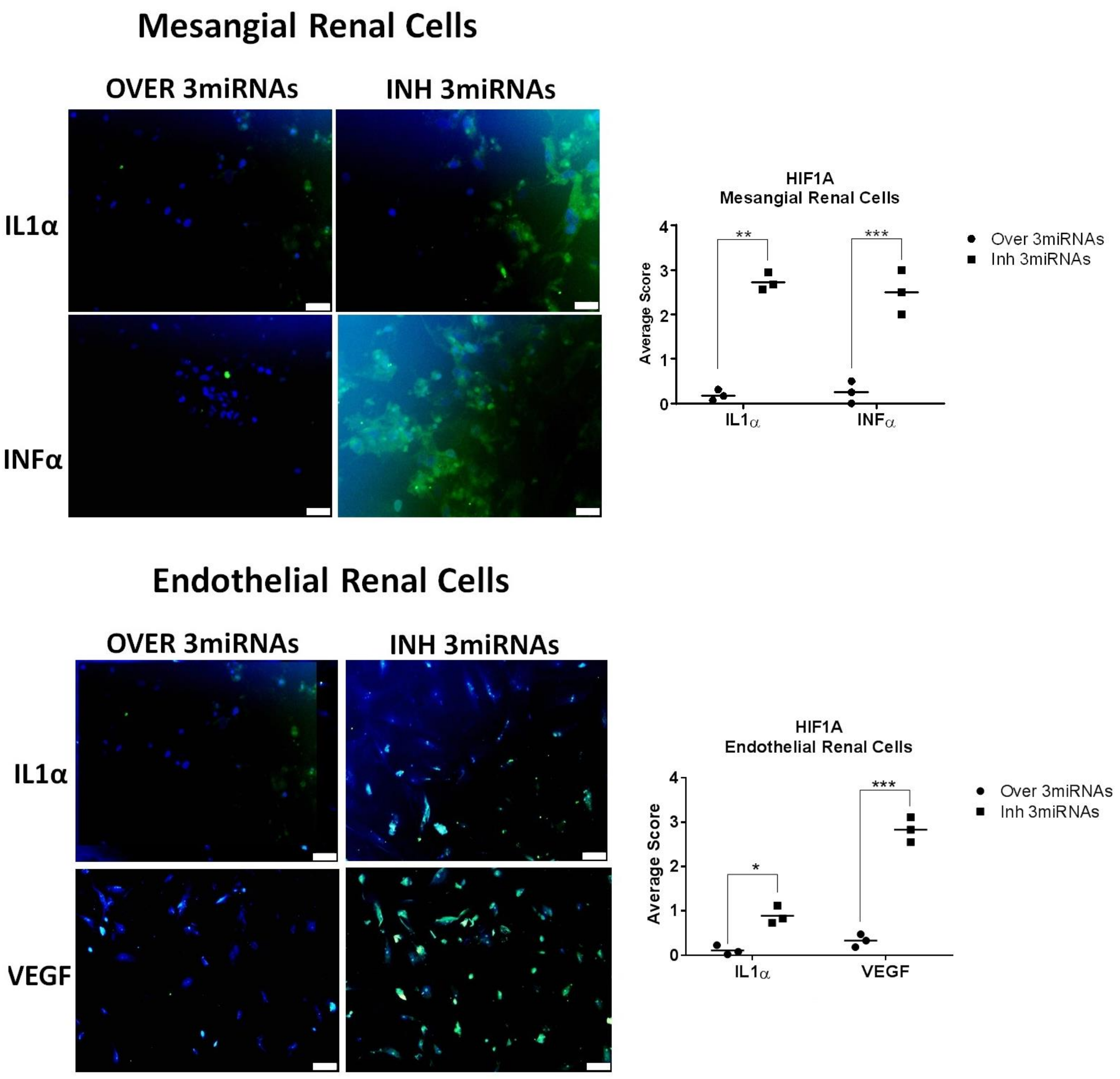

Figure S6. Protein levels of hypoxia-inducible factor 1-alpha (HIF1A) in mesangial and endothelial renal cells. Overexpression of the three microRNAs (miRNAs, miR-31, miR-107, and miR-135b-5) in both types of renal cells induced a significant reduction of HIF1A protein levels. *** $p < 0.0001$, ** $p < 0.001$, * $p < 0.01$. Scale bar = 20 μm.

4. Supplementary Table

Table S1. The MiRCURY LNA miRNA focus PCR Urine Exosome Panel I gene list for each 96-well plate.

MicroRNA Name	Order in 96 wells (row)	Order in 96 wells (column)	Panel Plate Position	Target Sequence	LNA PCR Primer Set, Product number	Assay Type
hsa-let-7a-5p	1	1	A01	UGAGGUAGUAGGUUGUAUAGUU	YP00205727	GOI
hsa-let-7b-5p	2	9	A02	UGAGGUAGUAGGUUGUGUGGUU	YP00204750	GOI
hsa-let-7d-3p	3	17	A03	CUAUACGACCUGCUGCCUUUCU	YP00205627	GOI
hsa-let-7d-5p	4	25	A04	AGAGGUAGUAGGUUGCAUAGUU	YP00204124	GOI
hsa-let-7e-	5	33	A05	UGAGGUAGGAGGUUGUAUAGUU	YP00205711	GOI

5p						
hsa-let-7f-5p	6	41	A06	UGAGGUAGUAGAUUGUAUAGUU	YP00204359	GOI
hsa-let-7g-5p	7	49	A07	UGAGGUAGUAGUUUGUACAGUU	YP00204565	GOI
hsa-let-7i-5p	8	57	A08	UGAGGUAGUAGUUUGUGCUGUU	YP00204394	GOI
hsa-miR-10a-5p	9	65	A09	UACCCUGUAGAUCCGAAUUUGUG	YP00204778	GOI
hsa-miR-10b-5p	10	73	A10	UACCCUGUAGAACCGAAUUUGUG	YP00205637	GOI
hsa-miR-15a-5p	11	81	A11	UAGCAGCACAUAAUGGUUUGUG	YP00204066	GOI
UniSp3 IPC	12	89	A12	0	YP02119288	IPC
hsa-miR-15b-5p	13	2	B01	UAGCAGCACAUCAUGGUUUACA	YP00204243	GOI
hsa-miR-16-5p	14	10	B02	UAGCAGCACGUAAAUAUUGGCG	YP00205702	GOI
hsa-miR-17-5p	15	18	B03	CAAAGUGCUUACAGUGCAGGUAG	YP02119304	GOI
hsa-miR-20a-5p	16	26	B04	UAAAGUGCUUAUAGUGCAGGUAG	YP00204292	GOI
hsa-miR-21-5p	17	34	B05	UAGCUUAUCAGACUGAUGUUGA	YP00204230	GOI
hsa-miR-22-3p	18	42	B06	AAGCUGCCAGUUGAAGAACUGU	YP00204606	GOI
hsa-miR-22-5p	19	50	B07	AGUUCUUCAGUGGCAAGCUUUA	YP00204255	GOI
hsa-miR-23a-3p	20	58	B08	AUCACAUUGCCAGGGAUUUCC	YP00204772	GOI
hsa-miR-23b-3p	21	66	B09	AUCACAUUGCCAGGGAUUACC	YP00204790	GOI
hsa-miR-24-3p	22	74	B10	UGGCUCAGUUCAGCAGGAACAG	YP00204260	GOI
hsa-miR-25-3p	23	82	B11	CAUUGCACUUGUCUCGGUCUGA	YP00204361	GOI
UniSp6 CP	24	90	B12	0	YP00203954	Spike
hsa-miR-26a-5p	25	3	C01	UUCAAGUAAUCCAGGAUAGGCU	YP00206023	GOI
hsa-miR-26b-5p	26	11	C02	UUCAAGUAAUUCAGGAUAGGU	YP00204172	GOI
hsa-miR-27a-3p	27	19	C03	UUCACAGUGGCUAAGUUCCGC	YP00206038	GOI
hsa-miR-27b-3p	28	27	C04	UUCACAGUGGCUAAGUUCUGC	YP00205915	GOI
hsa-miR-29a-3p	29	35	C05	UAGCACCAUCUGAAAUCGGUUA	YP00204698	GOI
hsa-miR-29b-3p	30	43	C06	UAGCACCAUUUGAAAUCAGUGUU	YP00204679	GOI
hsa-miR-29c-3p	31	51	C07	UAGCACCAUUUGAAAUCGGUUA	YP00204729	GOI
hsa-miR-30a-5p	32	59	C08	UGUAAACAUCCUCGACUGGAAG	YP00205695	GOI
hsa-miR-30b-5p	33	67	C09	UGUAAACAUCCUACACUCAGCU	YP00204765	GOI
hsa-miR-30c-5p	34	75	C10	UGUAAACAUCCUACACUCUCAGC	YP00204783	GOI
hsa-miR-30d-5p	35	83	C11	UGUAAACAUCCCCGACUGGAAG	YP00206047	GOI
UniSp3 IPC	36	91	C12	0	YP02119288	IPC
hsa-miR-30e-3p	37	4	D01	CUUUCAGUCGGAUGUUUACAGC	YP00204410	GOI
hsa-miR-30e-5p	38	12	D02	UGUAAACAUCCUUGACUGGAAG	YP00204714	GOI
hsa-miR-31-3p	39	20	D03	UGCUAUGCCAACAUAUUGCCAU	YP00204079	GOI

hsa-miR-31-5p	40	28	D04	AGGCAAGAUGCUGGCAUAGCU	YP00204236	GOI
hsa-miR-34a-5p	41	36	D05	UGGCAGUGUCUUAGCUGGUUGU	YP00204486	GOI
hsa-miR-92a-3p	42	44	D06	UAUUGCACUUGUCCCGGCCUGU	YP00204258	GOI
hsa-miR-93-5p	43	52	D07	CAAAGUGCUGUUCGUGCAGGUAG	YP00204715	GOI
hsa-miR-99a-5p	44	60	D08	AACCCGUAGAUCCGAUCUUGUG	YP00204521	GOI
hsa-miR-99b-5p	45	68	D09	CACCCGUAGAACCGACCUUGCG	YP00205983	GOI
hsa-miR-101-3p	46	76	D10	UACAGUACUGUGAUAACUGAA	YP00204786	GOI
hsa-miR-103a-3p	47	84	D11	AGCAGCAUUGUACAGGGCUAUGA	YP00204063	GOI
UniSp3 IPC	48	92	D12	0	YP02119288	Spike
hsa-miR-106a-5p	49	5	E01	AAAAGUGCUUACAGUGCAGGUAG	YP00204563	GOI
hsa-miR-106b-5p	50	13	E02	UAAAGUGCUGACAGUGCAGAU	YP00205884	GOI
hsa-miR-107	51	21	E03	AGCAGCAUUGUACAGGGCUAUCA	YP00204468	GOI
hsa-miR-125b-5p	52	29	E04	UCCCUGAGACCCUAACUUGUGA	YP00205713	GOI
hsa-miR-126-3p	53	37	E05	UCGUACCGUGAGUAAUAAUGCG	YP00204227	GOI
hsa-miR-128-3p	54	45	E06	UCACAGUGAACCGGUCUCUUU	YP00205995	GOI
hsa-miR-133a-3p	55	53	E07	UUUGGUCCCCUUCAACCAGCUG	YP00204788	GOI
hsa-miR-135b-5p	56	61	E08	UAUGGCUUUUCAUUCCUAUGUGA	YP00204130	GOI
hsa-miR-141-3p	57	69	E09	UAACACUGUCUGGUAAAGAUGG	YP00204504	GOI
hsa-miR-145-5p	58	77	E10	GUCCAGUUUUCCCAGGAAUCCCU	YP00204483	GOI
hsa-miR-148a-3p	59	85	E11	UCAGUGCACUACAGAACUUUGU	YP00205867	GOI
hsa-miR-148b-3p	60	93	E12	UCAGUGCAUCACAGAACUUUGU	YP00204047	GOI
hsa-miR-149-5p	61	6	F01	UCUGGCUCCGUGUCUUCACUCCC	YP00204321	GOI
hsa-miR-151a-5p	62	14	F02	UCGAGGAGCUCACAGUCUAGU	YP00204007	GOI
hsa-miR-181a-5p	63	22	F03	AACAUUCAACGCUGUCGGUGAGU	YP00206081	GOI
hsa-miR-187-3p	64	30	F04	UCGUGUCUUGUGUUGCAGCCGG	YP00204018	GOI
hsa-miR-191-5p	65	38	F05	CAACGGAAUCCCAAAAGCAGCUG	YP00204306	GOI
hsa-miR-193b-3p	66	46	F06	AACUGGCCCUCAAAGUCCCGCU	YP00204226	GOI
hsa-miR-195-5p	67	54	F07	UAGCAGCACAGAAAUAUUGGC	YP00205869	GOI
hsa-miR-197-3p	68	62	F08	UUCACCACCUUCUCCACCCAGC	YP00204380	GOI
hsa-miR-200a-3p	69	70	F09	UAACACUGUCUGGUAACGAUGU	YP00204707	GOI
hsa-miR-200b-3p	70	78	F10	UAAUACUGCCUGGUAAUGAUGA	YP00206071	GOI
hsa-miR-200c-3p	71	86	F11	UAAUACUGCCGGGUAAUGAUGGA	YP00204482	GOI
hsa-miR-203a	72	94	F12	GUGAAAUGUUUAGGACCACUAG	YP00205914	GOI

hsa-miR-204-5p	73	7	G01	UUCCCUUUGUCAUCCUAUGCCU	YP00206072	GOI
hsa-miR-210-3p	74	15	G02	CUGUGCGUGUGACAGCGGCUGA	YP00204333	GOI
hsa-miR-221-3p	75	23	G03	AGCUACAUUGUCUGCUGGGUUUC	YP00204532	GOI
hsa-miR-222-3p	76	31	G04	AGCUACAUCUGGCUACUGGGU	YP00204551	GOI
hsa-miR-301a-3p	77	39	G05	CAGUGCAAUAGUAUUGUCAAAGC	YP00205601	GOI
hsa-miR-342-3p	78	47	G06	UCUCACACAGAAAUCGCACCCGU	YP00205625	GOI
hsa-miR-365a-3p	79	55	G07	UAAUGCCCCUAAAAAUCCUUAU	YP00204622	GOI
hsa-miR-375	80	63	G08	UUUGUUCGUUCGGCUCGCGUGA	YP00204362	GOI
hsa-miR-378a-3p	81	71	G09	ACUGGACUUGGAGUCAGAAGG	YP00205946	GOI
hsa-miR-423-3p	82	79	G10	AGCUCGGUCUGAGGCCCCUCAGU	YP00204488	GOI
hsa-miR-423-5p	83	87	G11	UGAGGGGCAGAGAGCGAGACUUU	YP00205624	GOI
hsa-miR-425-5p	84	95	G12	AAUGACACGAUCACUCCCGUUGA	YP00204337	GOI
hsa-miR-429	85	8	H01	UAAUACUGUCUGGUAAAACCGU	YP00205901	GOI
hsa-miR-500a-5p	86	16	H02	UAAUCCUUGCUACCUGGGUGAGA	YP00204794	GOI
hsa-miR-532-5p	87	24	H03	CAUGCCUUGAGUGUAGGACCGU	YP00204221	GOI
hsa-miR-574-3p	88	32	H04	CACGCUCAUGCACACACCCACA	YP00206011	GOI
hsa-miR-582-5p	89	40	H05	UUACAGUUGUUCAACCAGUUACU	YP00204254	GOI
hsa-miR-598-3p	90	48	H06	UACGUCAUCGUUGUCAUCGUCA	YP00204320	GOI
hsa-miR-660-5p	91	56	H07	UACCCAUUGCAUAUCGGAGUUG	YP00205911	GOI
cel-miR-39-3p CP	92	64	H08	0	YP00203952	Spike
UniSp2 CP	93	72	H09	0	YP00203950	Spike
UniSp4 CP	94	80	H10	0	YP00203953	Spike
UniSp5 CP	95	88	H11	0	YP00203955	Spike
Blank (H20)	96	96	H12	#N/A	Empty	Empty

Table S2. MiRCURY LNA primer identification (IDs) from Exiqon.

MiRNAs	Primer ID
hsa-miR-17-5p	204771
hsa-miR-20a-5p	204292
hsa-miR-423-3p	204488
hsa-miR-365a-3p	204622
hsa-miR-10a-5p	204778
hsa-miR-151a-5p	204007
hsa-miR-204-5p	206072
hsa-let-7i-5p	204394
hsa-miR-31-5p	04079
hsa-miR-195-5p	205869
hsa-miR-15a-5p	204066
hsa-miR-107	204468
hsa-miR-532-5p	204221
hsa-miR-29a-3p	204698
hsa-miR-378a-3p	205946

hsa-miR-135b-5p	204130
hsa-miR-203a	205914

Table S3. Primers identification (IDs) used in Taqman reverse transcription polymerase chain reaction (RT-PCR) from Applied Biosystems.

Gene	Primer ID
CXCL1	Hs00236931_m1
CCL2	Hs00234140_m1
CCL3	Hs00234142_m1
IL6	Hs00174131_m1
IL8 (CXCL8)	Hs00174103_m1
VCAM1	Hs01003372_m1
HIF1A	Hs00153153_m1
FN1	Hs01549976_m1
COL3A1	Hs00943809_m1
TGFβ1	Hs00998133_m1
GAPDH	Hs02786624_g1

Table S4. MicroRNAs ordered according to increasing *p*-value in each of the analyses. MiRNAs marked in bold were selected for the validation phase (fold change >|10| and *p*-value < 0.05). RQ = relative quantification-$2^{-\Delta\Delta Ct}$; $\Delta\Delta Ct = \Delta Ct_{sample} - \Delta Ct_{calibrator}$.

**(1) Responders vs Non-Responders at Flare Time (A1 vs B1)**		
**microRNA**	**Fold Change**	***p* Value**
**miR-10a-5p**	-220.5003	0.02369
**miR-151a-5p**	-231.4115	0.02419
**miR-204-5p**	-245.0021	0.02971
**let-7i-5p**	-261.8712	0.04722
**(2) Responders vs Non-Responders at Post-Flare Time (A2 vs B2)**		
**microRNA**	**Fold Change**	***p* Value**
**miR-365a-3p**	-528.5379	0.03751
**(3) Post-Flare vs Flare Time in the Responder group (A1 vs A2)**		
**microRNA**	**Fold Change**	***p* Value**
**miR-107**	10.14259	0.01792
**miR-203**	28.96633	0.01808
**miR-378a-3p**	13.32	0.02088
miR-17-5p	8.15	0.02178
let-7b-5p	8.92	0.02436
**miR-195-5p**	93.47	0.02982
miR-532-5p	8.59	0.03533
miR-29a-3p	4.875	0.03548
**miR-29b-3p**	22.9483	0.03713
**miR-135b-5p**	16.6869	0.03769
miR-598	9.01968	0.04023
**miR-31-5p**	13.2852	0.0418
**(4) Post-Flare vs Flare time in the Non-Responder Group (B1 vs B2)**		
**microRNA**	**Fold Change**	***p* Value**
miR-26b-5p	2.2894	0.00055
**miR-15a-5p**	104.17213	0.00398
miR10a-5p	-6.43045	0.01734
miR-31-3p	3.49583	0.0199

miR-22-5p	3.50865	0.02431
**miR-148-3p**	-155.6842	0.029
**miR-99b-5p**	-14.8411	0.03883
**miR-31-5p**	13.2852	0.0418
miR-195-5p	9.83608	0.04394
**miR-25-3p**	-170.5254	0.04952

Table S5. Target genes for miR-31, miR-107, and miR-135b-5p using the miRecords, miRTarBase, and miRWalk databases. Underlined targets are common between two microRNAs (miRNAs).

MiRNA	Described Target Genes
**MiR-31**	*AG001, ABCB9, ACBD7, AFF1, AKAP8L, AKNA, AR, ARF1, ARID1A, ARPC5, ATP2A2, ATP5A1, BAHD1, BCAS4 C11orf30, C15orf52, C19orf12, C2CD5, <u>CASR</u>, CCDC127, CCNT1, CDC42SE1, CDK1, CKAP2L, CNBP, CREG1, CXCL12 CYP27B1, DACT3, DDX19A, DKK1, DMD, DNAJC5, DPM2, DPYSL5, ECHDC1, EDC3, ETS1, EXOC6, EXOSC5 FAM134C, FAM193A, FLNA, FOXC1, FOXD4, FOXD4L1, FOXD4L4, FOXD4L5, FOXP3, FRK, FZD1, FZD3, GHITM, GLI2 GTF2E1, GUF1, GYG1, HEATR2, HIF1AN, HIST1H2BC, HIST1H2BJ, HIST1H2BK, HOXA7, HOXC13, HOXD3, HOXD3, ICAM1, IL5RA, ILF2, INTU, IPP, ITGA5, JARID2, JAZF1, KLF13, KLHL15, LAPTM4A, <u>LATS2</u>, LILRA2, LIPG, LRRC59, MAP4K4, MCM2, MCMBP, MED12, MET, MICA, MLH1, MMP16, MPRIP, MXRA7, MYO1D, MZT1, NDFIP2, NF2, NFAT5, NFATC2IP, NOL9, NOP56, NUDT3, NUMB, NUP188, PAPOLG, PARP1, PARP11, PCSK1N, PEX19, PHF12, PPIL2, PPM1L, PPP2R2A, <u>PRKCE</u>, PTPRJ, PYURF, QSER1, RAB27A, RANGAP1, RASA1, RDX, RET, REXO2, RHOA, RHOBTB1, RNF111, RPA1, RPL12, RPL27A, RPL35A, RPL37A, RPS4Y1, RPS7, RSRC1, SATB2, SDC4, SELE, SESN2, SFT2D2, SFXN1, SLC18B1, SMEK2, SMG1, SNRNP27, SP1, SP7, SPRTN, SRC, SRPX2, SRRM2, STK40, STOML2, SYDE2, TBXA2R, TIAM1, TMEM109, TNRC6B, TOR1AIP1, TRIB3, TRRAP, TSPAN1, TXNDC5, UBA6, WASF3, XPO6, YWHAE, YY1, ZC3H12C, ZC3H18, ZDHHC6, ZIK1, ZNF275, ZNF331, ZNF460, ZNF587, ZNF641, ZNF678, ZNF805*
**MiR-107**	*AGO01, AGO02, AGO03, ABCF2, ABL2, ACTR2, ACVR2B, ADD2, ADORA3, ALDH3B1, AMOT, ANKFY1, ARGLU1, ARIH1, ARNT, ASH1L, ATG12, ATG14, ATP13A3, ATXN1L, AXIN2, B3GNT2, BABAM1, BACE1, BAZ2A, BTLA, C16orf72, C21orf58, C9orf62, CAB39, CALU, CAPZA2, CAV1, CCDC83, CCNE1, CCNT1, CD180, CDADC1, CDC42SE2, CDCA4, CDK1, CDK6, CDK8, CDV3, CHRM1, CKMT1A, CLIP1, CNNM2, COPS7A, CPEB3, CREBRF, CRKL, CSNK1G3, CSNK2A1, CYP2C8, CYSLTR2, DAPK1, DECR1, DEPDC1B, DHX16, DHX33, DICER1, DMPK, DNAJA1, DNAJC10, DOCK11, DST, DUSP14, DYRK2, EFTUD2, EI24, EIF1AX, ELK4, EML4, EN2, ENPP2, ENTPD1, ERN1, FAM103A1, FAM207A, FAM229B, FAM49A, FAM98A, FAM9C, FBXW7, FCF1, FGF2, FGFRL1, FOXC1, <u>FOXO1</u>, FURIN, FZD6, G3BP2, GABRB1, GANAB, GCC1, GLP2R, GNAT1, GNG12, GNS, GOLGA8B, GPCPD1, GPR89B, GPRC5A, GRN, GSG1, GUCD1, HCFC2, HDDC2, HIC2, <u>HIF1A</u>, HNRNPA2B1, HPRT1, IDH3A, IER3IP1, IGSF3, IL6, INSIG1, ITGA2, JAK1, JOSD1, KDELR1, KIF23, <u>KLF4</u>, L2HGDH, <u>LATS2</u>, LBR, LCOR, LIN7C, LRIF1, LUC7L, MAP3K7, MCM7, METRNL, MIS18BP1, MPDU1, MPLKIP, MRPL12, MRPL2, MRPL51, MT1E, MTFR1L, MTMR3, MYB, MYBPC1, N4BP1, NACC2, NFIA, NINJ1, NNT, NOTCH2, NPY4R, NUCKS1, NUMB, NUP50, NUS1, ODF2L, OGT, OPRM1, ORC4, OTUD7B, PAFAH1B2, PAG1, PAWR, PCSK5, PDZD8 PER1, PHGDH, PHKA1, PIK3R1, PLAG1, PLEKHA1, PLEKHF2, PNISR, POLD3, POLDIP2, PPIG, PPIL1, PPP1R16B, PPP2CA, PPP2R5C, PPP6C, PPP6R3, <u>PRKCE</u>, PRR14L, PSMB6, PTEN, PURB, RAB10, RAB1B, RACGAP1, RAD21, RAD51, RBBP6, RCC1, REL, RIMS3, RNF168, RPAIN, RPL27, RPS24, RPS6KB1, RPSA, RRAGC, RS1, RSL1D1, RUNX1T1, SALL1, SALL4, SAT1, SEMA6A, SETD1B, SLAIN2, SLC28A1,*

	SLC2A3, SLC30A7, SLCO3A1, SMARCA5, SMARCE1, SNCG, SNTB2, SNTB2, SNX8, SOWAHC, SREK1, SRSF1, SSU72, STK38, STX6, SUN2, SUN2, SYNRG, TAF13, TARBP2, TBPL1, TBRG1, TCF19, TGFBR3, TJP1, TK1, TM4SF1, TM7SF3, TM9SF1, TMEM170A, TMEM87A, TNRC6B, TRIM35, TRIQK, TSC22D2, TTLL5, TUBA1B, TUBB, TULP4, TWF1, UBE2B, UBE2Q1, UBR3, USP42, VCAN, VEGFA, VPS4A, WDR6, YAF2, YIPF6, YRDC, YTHDC1, YWHAH, ZADH2, ZBTB10, ZBTB38, ZCCHC14, ZDHHC16, ZDHHC4, ZNF100, ZNF273, ZNF449, ZNF585B, ZNF606, ZNF680
**MiR-135b-5p**	*ACVR1B, AMOTL2, APC, APOA1, ARC, BGLAP, BIRC5, BMPR2, CAPZA2, CASR, CCDC85C, CDR1, CENPN, CEP135, COX6B1, DPP8, FOXO1, GAGE1, GATA6, GNL1, HEYL, HIF1A, HMGB2, IBSP, IL17RA, KIAA1143, KIF6, KLF4, LAX1, LRRC15, LZTS1, MAFB, MARCKS, MBNL1, MID1, MPL, MTCH2, MYC, NHS, NSA2, NUFIP2, PCP4L1, PCTP, PEX2, PIAS4, PIP5K1A, RAB3GAP2, RUNX2, SCYL3, SKIL, SLC19A3, SLC39A6, SMAD5, SNED1, SP7, TGFBR1, TNPO2, TOX4, TRIM4, TRIM66, TTLL7, TXNIP, ZNF107, ZNF468, ZNF805, ZNF846*
